# Phage Display and Synthetic Peptides as Promising Biotechnological Tools for the Serological Diagnosis of Leprosy

**DOI:** 10.1371/journal.pone.0106222

**Published:** 2014-08-29

**Authors:** Silvana Maria Alban, Juliana Ferreira de Moura, Vanete Thomaz-Soccol, Samira Bührer Sékula, Larissa Magalhães Alvarenga, Marcelo Távora Mira, Carlos Chávez Olortegui, João Carlos Minozzo

**Affiliations:** 1 Department of Biotechnology and Bioprocess Engineering, Federal University of Parana, Curitiba, Parana, Brazil; 2 Basic Pathology Department, Federal University of Parana, Curitiba, Parana, Brazil; 3 Immunology Department, Tropical Pathology and Public Health Institute, Federal University of Goias, Goiania, Goias, Brazil; 4 Health Sciences Postgraduate Program, School of Medicine, Pontifical Catholic University of Parana, Curitiba, Parana, Brazil; 5 Department of Biochemistry and Immunology, Federal University of Minas Gerais, Belo Horizonte, Minas Gerais, Brazil; 6 Center for Production and Research of Immunobiological Products, Parana State Department of Health, Piraquara, Parana, Brazil; Federal University of São Paulo, Brazil

## Abstract

**Background:**

The diagnosis of leprosy is primarily based on clinical manifestations, and there is no widely available laboratory test for the early detection of this disease, which is caused by *Mycobacterium leprae*. In fact, early detection and treatment are the key elements to the successful control of leprosy.

**Methodology/Principal Findings:**

Peptide ligands for antibodies from leprosy patients were selected from phage-displayed peptide libraries. Three peptide sequences expressed by reactive phage clones were chemically synthesized. Serological assays that used synthetic peptides were evaluated using serum samples from leprosy patients, household contacts (HC) of leprosy patients, tuberculosis patients and endemic controls (EC). A pool of three peptides identified 73.9% (17/23) of multibacillary (MB) leprosy patients using an enzyme-linked immunosorbent assay (ELISA). These peptides also showed some seroreactivities to the HC and EC individuals. The peptides were not reactive to rabbit polyclonal antisera against the different environmental mycobacteria. The same peptides that were conjugated to the carrier protein bovine serum albumin (BSA) induced the production of antibodies in the mice. The anti-peptide antibodies that were used in the Western blotting analysis of *M. leprae* crude extracts revealed a single band of approximately 30 kDa in one-dimensional electrophoresis and four 30 kDa isoforms in the two-dimensional gel. The Western blotting data indicated that the three peptides are derived from the same bacterial protein.

**Conclusions/Significance:**

These new antigens may be useful in the diagnosis of MB leprosy patients. Their potentials as diagnostic reagents must be more extensively evaluated in future studies using a large panel of positive and negative sera. Furthermore, other test approaches using peptides should be assessed to increase their sensitivity and specificity in detecting leprosy patients. We have revealed evidence in support of phage-displayed peptides as promising biotechnological tools for the design of leprosy diagnostic serological assays.

## Introduction

Leprosy, which is caused by *Mycobacterium leprae* [1], remains a currently relevant disease. As of 2012, cases were reported in 115 countries with the vast majority being concentrated in India, Brazil, and Indonesia [2]. Despite the reduction in the global prevalence rate from 5.4 million in 1985 to 181,941 at the beginning of 2012, the number of new cases that are detected has remained stable over recent years [2], indicating the continuity of its transmission [3].

The clinical manifestations are determined by the immune response of the patient to *M. leprae*. Individuals with lepromatous leprosy present with high bacterial loads and exacerbated humoral immune responses. In contrast, those with tuberculoid leprosy show few bacilli in their lesions and intense cellular immune responses that can be evaluated by the lepromin test [1], [4]. Most patients present borderline leprosy clinical forms [1]. The diagnosis of this disease is essentially clinical and is occasionally accompanied by bacteriological or histological examinations [5]. With regard to treatments, the World Health Organization (WHO) has proposed clinical classifications, including the numbers of skin lesions and nerves that are involved, for grouping the leprosy patients into MB and paucibacillary (PB) categories [6]. Patients with up to five lesions are classified as PB, and those with more than five lesions are classified as MB. However, classifications that are based solely on the numbers of lesions impair the proper diagnosis of this disease. Many MB populations with few skin lesions are incorrectly classified as PB; therefore, they are inadequately treated and run the risk of relapse [7].

Regarding immunological diagnostic assays, the presence of antibodies against phenolic glycolipid I (PGL-I) has been extensively studied. While anti-PGL-I serology can detect the majority of MB patients, it has limited value in identifying PB patients. In addition, false positive results in the areas of endemicity are relatively high (>10%) [8], [9]. Consequently, it has been recommended that PGL-I-based tests be used in support of the clinical examinations to direct the clinicians towards appropriate treatment and none of these PGL-I-based tests have been widely implemented in field situations [10]. Thus, tools that permit the correct and early diagnosis of *M. leprae* infection in patients who are displaying symptoms are a priority in leprosy research. The search for antigens for immunological diagnoses was initially based on research using total extracts and subcellular fractions of *M. leprae* followed by advances that were achieved using recombinant DNA technology and, more recently, on studies involving comparative genomic analyses and bioinformatics. The main difficulty that is encountered involves obtaining reagents that are more sensitive and specific or that distinguish *M. leprae* exposure from infection. The low specificity of the antigens is a result of cross-reactivity with other mycobacteria, which becomes even more problematic in countries with high incidence rates of tuberculosis and routine *M. bovis* bacillus Calmette-Guerin (BCG) vaccinations [11].

Accordingly, this study proposes the use of the phage-display technique as a tool to identify new reagents that may be effectively used in immunological assays. We have extended our previous observations by evaluating the potentials of peptide mimotopes of *M. leprae* antigens selected by the screening of phage-displayed random peptide libraries as potential serological test reagents for leprosy diagnosis. The mimotopes were tested on leprosy patients, HC, EC, tuberculosis patients and with immune sera that were raised against several mycobacteria in animals. The results indicate that peptide mimotopes may be useful for the diagnosis of leprosy.

## Methods

### Ethics statement

All of the animal care and experimental procedures and human blood sample collections were performed in accordance with the institutional guidelines. All of the individuals provided written informed consent prior to venipuncture. The animal protocols were conducted in compliance with the Brazilian rules of animals used for experimental purposes. These protocols are based on national and international guidelines, such as those that have been disclosed by the following organizations: the International Guiding Principles for Biomedical Research Involving Animals (CIOMS), the American Association for Laboratory Animal Science (AALAS), and the Brazilian College of Animal Experimentation (COBEA).

Both animal procedure and experiment involving human subjects were approved by the Research Ethics Committee of the Federal University of Parana (UFPR), Curitiba, Brazil (protocol number CEP/SD 428.108.07.10).

### Human sera

The leprosy patients and HC were recruited from the Clinical Dermatology Hospital of Parana (Piraquara, Brazil), the Barão Regional Specialties Center (Curitiba, Brazil), the Pro-Hansen Foundation (Curitiba, Brazil), and the Humanitas Philanthropic Society (São Jerônimo da Serra, Brazil). Sera from 10 PB and 23 MB leprosy patients were included, of whom 14 were newly diagnosed and 19 had received up to four months of treatment. In addition, sera were included from 26 HC, 30 patients with pulmonary tuberculosis (TB) who had been treated for up to three months in specialized centers, and 30 healthy EC with no known previous exposure to *M. leprae* or *M. tuberculosis*.

### Synthetic phage library derived-peptides evaluated by ELISA assay

Procedures for the biopanning of phage libraries with the anti-*M. leprae* antibodies of MB patients were performed as previously described [12], [13]. The peptide sequences in the reactive phage clones were synthesized using a 9-fluorenylmethoxycarbonyl (Fmoc)-based solid-phase synthesis technique.

The ELISA test was optimized with regard to antigen concentration and serum and conjugate dilutions. Microtiter plates (Corning) were coated with 100 µL of the peptide pool at 1.5 µg/mL in 0.05 M carbonate buffer (pH 9.6) overnight at 4°C. After washing with a solution containing 0.9% NaCl and 0.05% Tween 20, the plates were blocked with Protein-Free Blocking Buffer (Thermo Fisher Scientific) for 1 h at 37°C. Then, the plates were washed and incubated for 1 h at 37°C with sera in duplicate dilutions of 1∶50 in a phosphate-buffered saline (PBS) solution at pH 7.4, which also contained 0.1% BSA. The plates were washed and incubated with anti-human IgG (Fc-specific)-biotin antibodies (Sigma-Aldrich) that were diluted 1∶2 500 in PBS with 0.1% BSA for 1 h at 37°C. The plates were then washed and incubated with streptavidin-peroxidase (Sigma-Aldrich) that was diluted 1∶2 500 in PBS with 0.1% BSA for 1 h at 37°C and detected using ο-phenylenediamine (OPD) dihydrochloride (Sigma-Aldrich). The reactivities of the rabbit anti-mycobacterial sera with the peptides were also analyzed (data not shown). In this analysis, the antibodies that were bound to the peptides were detected by anti-rabbit IgG-peroxidase antibodies that were diluted to 1∶4 000.

### Immunoassay with cellulose membrane-bound peptides

The peptides and their alanine analogs were prepared by Spot Synthesis [14]. Peptide synthesis was performed using Fmoc-protection chemistry on cellulose membranes that had been derivatized with polyethylene glycol spacers (Intavis Bioanalytical Instruments AG) and MultiPep RS (Intavis Bioanalytical Instruments AG). Following overnight incubation with a blocking solution (0.05% phosphate buffered saline tween-20 (PBST) and 3% BSA), the membrane was probed with the sera of the MB patients at 1∶250 dilutions in PBST for 1 h at room temperature. The reaction was developed with the anti-human IgG (Fc-specific)-peroxidase antibody that was diluted to 1∶120 000 in PBST for 1 h in addition to ECL Plus (GE Healthcare) and Hyperfilm ECL (GE Healthcare). The spot intensities were determined using the ImageJ software (version 1.43).

### Research of native proteins in *M. leprae* using anti-peptide antibodies

The synthetic peptides were conjugated to BSA using glutaraldehyde as a cross-linker [15]. Conjugates (20 µg) that were emulsified in incomplete Freund's adjuvant (Sigma-Aldrich) were administered via subcutaneous (first and second doses) and intraperitoneal (third to fifth doses) injections to eight-week-old Swiss female mice (n = 5 per group). The injections were performed on days 1, 14, 28, 42, and 56. Non-immune serum was used as the negative control, and immune blood was collected one week after the final dose. To assess the production of anti-peptide antibodies, microtiter plates (Corning) were coated with 100 µL of the peptide at final concentrations of 1.5 µg/mL in 0.05 M carbonate buffer (pH 9.6) overnight at 4°C. After washing in a solution containing 0.9% NaCl and 0.05% Tween 20, the plate was blocked (2% casein in PBS, pH 7.4) for 1 h at 37°C. The plate was then washed and incubated for 1 h at 37°C with sera that had been diluted in incubation buffer (0.25% casein in 0.05% PBST). After washing, the detection of the reaction was performed using the anti-mouse IgG (γ-specific)-peroxidase antibody (Sigma-Aldrich) at a 1∶2 500 dilution and OPD dihydrochloride as a chromogen.

The *M. leprae* whole cells were provided by Dr. J. S. Spencer from Colorado State University, Fort Collins, USA through the National Institute of Allergy and Infectious Diseases/National Institutes of Health under the contract N01-AI-25469 for the production of soluble antigens for use in electrophoresis. The cells were resuspended in 0.9% NaCl, mixed with 100 µg/mL phenylmethanesulfonyl fluoride (PMSF) and 2 mM ethylenediaminetetraacetic acid (EDTA) and sonicated (Sonopuls HD 2200) for four cycles of 15 min each [16]. The protein concentration of the soluble fraction that was obtained by centrifugation at 10 000 *g* for 20 min at 4°C was determined using the Quant-iT Protein Assay Kit (Invitrogen). *M. leprae* protein (40 µg) were then electrophoresed on a 15% sodium dodecyl sulfate-polyacrylamide gel electrophoresis (SDS-PAGE) and transferred to a PVDF membrane that was blocked with 0.3% PBST, washed with 0.05% PBST and incubated with anti-peptide serum in 0.05% PBST. The membrane was washed, incubated with anti-mouse IgG (γ-specific)-peroxidase antibodies that were diluted to 1∶5 000 in 0.05% PBST, and processed using ECL Plus.

For the identification of the proteins in *M. leprae*, 2-dimensional gel electrophoresis (2-DE) followed by Western blotting were performed. Two hundred µg of *M. leprae* protein were purified using a 2D Clean-Up Kit (GE Healthcare) and solubilized in rehydration buffer (8 M urea, 2% CHAPS, 20 mM DTT, 0.5% IPG buffer, and 0.002% bromophenol blue). Samples were in gel rehydrated and run on linear 13 cm IPG strips at pH levels ranging from 4–7 (GE Healthcare) with the Ettan IPGphor II (GE Healthcare) for 21400 Vh. The focused strips were equilibrated in a solution that contained 6 M urea, 50 mM Tris (pH 8.8), 30% glycerol, 2% SDS, 0.002% bromophenol blue and 0.065 M DTT for 15 min, followed by incubation in the same solution that was prepared with 0.14 M iodoacetamide instead of DTT for an additional 15 min. The second-dimensional separation was performed using 15% SDS-PAGE by the SE 600 Ruby (GE Healthcare) electrophoresis unit. Western blotting was performed as previously described.

### Statistical analyses

A cut-off point for optimal sensitivity and specificity for the ELISA tests was determined using the Receiver Operating Characteristic (ROC) curve analysis [17], [18]. The accuracy of each test was evaluated according to the area under the curve (AUC) and the Youden index J [19]. The statistical analyses were performed using the MedCalc statistical software (version 13.2.0).

The differences in seroreactivities between the groups were analyzed with the two-tailed Mann-Whitney U test for nonparametric distribution using the GraphPad Prism software (version 5.03). The *P* values were corrected for multiple comparisons. The *P* value indicating statistical significance was set to <0.05.

## Results

### Peptide sequences

Table 1 shows the peptide sequences that were selected by phage display using the affinity purified anti-*M. leprae* MB patients' antibodies as previously reported [12]. The consensus sequence DPAW was observed between peptides 5A and 6A.

**Table 1:** Amino acid sequences of the peptides obtained by phage display.

Peptide	Amino acid sequence	Length
5A	APDDPAWQNIFNLRR	15
6A	ICPRDPAWSYCN	12
1B	NNIHAHRYWGTDLNA	15

### ELISA using synthetic peptides

**Figure 1:**
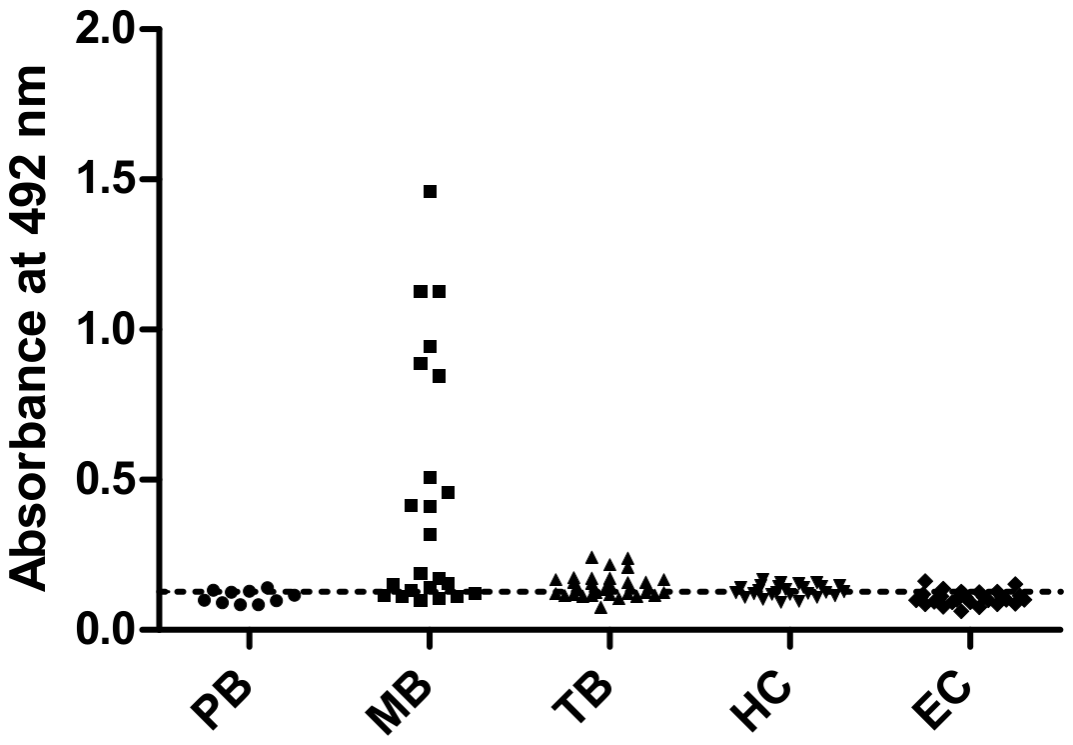
ELISA reactivities of synthetic peptides with sera from leprosy patients and controls. Microtiter plates were coated with 1.5 µg/mL of the peptide pool (5A, 6A, and 1B) and incubated with serum that was diluted 1∶50. The detection of the reaction was performed with the anti-human IgG (Fc-specific)-biotin antibody and streptavidin-peroxidase. MB, MB leprosy patients (n = 23); PB, PB leprosy patients (n = 10); TB, TB patients (n = 30); HC, healthy household contacts of MB leprosy patients (n = 26); EC, endemic controls (n = 30). The dotted line represents the cut-off value (as determined using the ROC curve with serum samples from the EC). Each symbol represents the absorbance obtained with a single serum.

The peptides 5A, 6A, and 1B were chemically synthesized and evaluated by ELISA against the sera from the leprosy patients. The peptides were capable of detecting 30% (3/10) of the PB and 73.9% (17/23) of the MB patients (Table 2). Compared with the PB leprosy patients, MB patients, in general, showed strong positive responses. Of the 30 serum samples from the tuberculosis patients, 16 (53.3%) were reactive to the peptides (Table 2). Weak positive responses were observed in 12 out of 26 of the HC sera and in 5 out of the 30 EC sera that were tested in this experiment (Table 2 and Fig. 1). These findings indicate the existence of significant differences between the leprosy (PB and MB groups) and EC groups and the TB and EC groups. No significant differences were observed between HC and EC groups. The sensitivity, specificity, AUC and Youden index J values were calculated for ELISA assay (Table 3). The peptides were not reactive against the rabbit antimycobacterial sera using the ELISA (data not shown).

**Table 2:** ELISA results using peptides in the groups studied.

Group	Number of serum samples tested	Number (%) of positive serum samples by ELISA with peptides
PB	10	3 (30)
MB	23	17 (73.9)
TB	30	16 (53.3)
HC	26	12 (46.2)
EC	30	5 (16.7)

MB: MB leprosy patients. PB: PB leprosy patients. TB: TB patients. HC: healthy household contacts of MB leprosy patients. EC: endemic control.

**Table 3:** Diagnostic performance of ELISA using peptides for the diagnosis of leprosy.

Serological test	Sensitivity in PB+MB groups % (95% CI)	Specificity in PB+MB groups % (95% CI)	AUC	Youden index J
ELISA-Peptides	60.6 (42.1–77.1)	90.0 (73.5–97.9)	0.779	0.5061

Samples from leprosy patients (n = 33, PB+MB leprosy patients) and endemic control (n = 30) were tested. CI: confidence interval. AUC: area under the ROC curve.

### Identification of key residues for antibody binding in peptides

**Figure 2:**
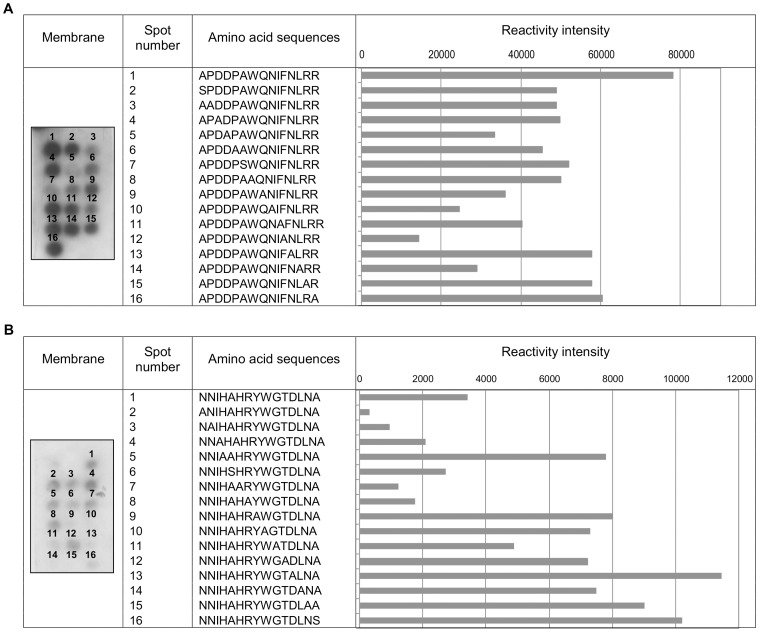
Identification by alanine scanning of key residues in peptides. Reactivities of antibodies from leprosy patients (1∶250) with membrane-bound peptides detected using the anti-human (Fc-specific)-peroxidase antibody (1∶120000). (A) The reference peptide 5A is in position 1, and the other spots correspond with the alanine analogs of peptide 5A. (B) The reference peptide 1B is in position 1 followed by its alanine analogues. Each bar represents the intensity of the antibody binding to the reference peptide or an analog peptide. When alanine was present in the generation position of the analog, the alanine residue was substituted by serine.

For peptide 5A, the proline, aspartate and alanine residues were important for antibody recognition. The substitution of these amino acids by alanine or serine reduced the reactivity of the peptide compared with that of the unmodified peptide. No reactivity was observed for peptide 6A. For peptide 1B, the residues of greatest importance to the antibody interactions were tyrosine, tryptophan, glycine, aspartate, leucine and asparagine (Fig. 2).

### Identification of natural antigens in *M. leprae*

**Figure 3:**
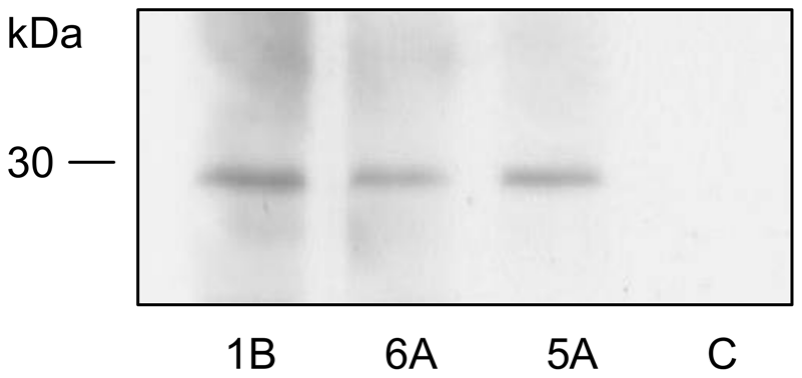
Reactivity of anti-peptide antibodies to *M. leprae* by Western blotting. Forty micrograms of total *M. leprae* extract were separated by 15% SDS-PAGE and, following Western blotting, were reacted with anti-peptide serum 1B (1∶12000), anti-peptide serum 6A (1∶9000), anti-peptide serum 5A (1∶4000), and non-immune serum (C) (1∶4000). The detection of the reaction was performed with the anti-mouse IgG (γ-specific)-peroxidase antibody and chemiluminescence.

**Figure 4:**
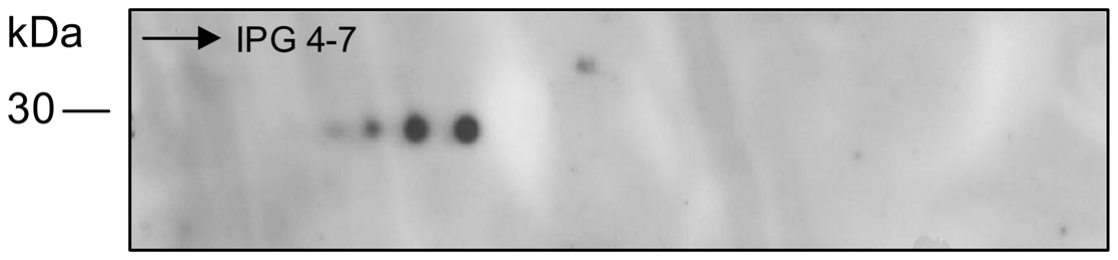
Western blotting analysis of total *M. leprae* extract 2D gel using mouse anti-peptide antibodies. Two hundred micrograms of protein were separated in the first dimension by isoelectric focusing on a 13 cm IPG strip at pH 4-7 and in the second dimension by SDS-PAGE on a 15% gel. Western blotting with anti-peptide serum 1B at 1∶4000 is shown. The other two anti-peptide sera showed the same reactivity profiles as that of anti-peptide serum 1B (data not shown). The detection of the reaction was performed with the anti-mouse IgG (γ-specific)-peroxidase antibody and chemiluminescence.

The peptides that were conjugated to BSA induced mouse anti-peptide antibody production and these antibodies reacted with antigens of *M. leprae* after electrophoresis, which was followed by Western blotting (Fig. 3). The Western blotting of the *M. leprae* proteins that was resolved by 2 DE and probed with the anti-peptide antibody revealed four spots of approximately 30 kDa in size (Fig. 4). The three anti-peptide antisera showed the same reactivity profiles with *M. leprae* (data not shown). The estimated isoelectric points (pI) for the four spots were 4.56, 4.64, 4.75 and 4.9.

### *In silico* analyses of peptides

The three peptide sequences that were identified demonstrated significant alignments with the antigen 85B (fbpB) of *M. leprae* using BLASTp. Antigen 85B is a member of the antigen 85 complex. It has a molecular mass of 31 kDa and a theoretical isoelectric point of 4.9.

## Discussion

In this study we evaluated the serological responses to peptides that were selected from random peptide phage-display libraries in an attempt to identify antigens that could be useful for the serological diagnoses of leprosy patients. The MB leprosy patients were easier to identify and the ELISA showed positive results in 73.9% of the samples that were tested. For the PB leprosy patients, 30% of the serum samples were reactive against the peptides as shown by the peptide-based ELISA and weaker responses were observed in this group compared with the MB group.

Protein antigens require binding to human leukocyte antigen (HLA) molecules to be presented by T cells. Because responses to mycobacterial antigens are HLA-restricted, not all individuals respond to the same antigens [20]. Reasons other than HLA restriction exist that explain the seronegativities between the patients that have been diagnosed with MB leprosy by the peptide-based ELISA. Recent research suggests that for tuberculosis, heterogeneous antigen recognition may result from the production of different antigens at different stages of the disease [21]. Another reason is related to differences in the strains of *M. leprae* [8]. However, research has shown that different strains have highly conserved genomes, which indicates that antigenic variations are likely to be negligible [22]. Because of the differences in the immune responses that occur among individuals, it has been suggested that immunological tests should be based on a combination of several different antigens to permit greater coverage in individuals that have been infected with *M. leprae* [3], [23].

The detection of *M. leprae* infection in PB patients through serological assays is difficult because once the immune response develops in these individuals, it is predominantly cellular. Analyses of T cell responses as interferon-gamma (IFN-γ) release assays are required for the diagnosis of PB [9]. Thus, the immunological diagnosis of leprosy may be based on both antibody and T cell assays [24]. The combination of these assays would permit the correct classification of patients and consequently, the initiation of effective treatments that may improve the prognoses for each case.

Seroreactivities against peptides were observed in the tuberculosis and HC and EC individuals. The recognition of peptides by tuberculosis patient sera was expected because *M. leprae* shares antigens with *M. tuberculosis*. Moreover, the diagnosis of leprosy or tuberculosis does not rely on a single test but must be confirmed by a combination of several tests and further associated with a clinical examination.

The capacity of the peptide-based ELISA test to predict disease development in the HC patients was not possible in this work because it requires that they be monitored over a prolonged period of time. Seroreactivity with some endemic control samples suggests that peptides not are capable of discriminate infection of exposure to *M. leprae*. Further investigation needs to be conducted testing the peptides with non-endemic controls to clarify this issue.

The understanding of the molecular basis of peptide recognition by patient antibodies may assist in the design of novel peptides with improvements in specificity and sensitivity for diagnostic purposes. The contribution of each amino acid residue in the peptide sequence to antibody recognition was deduced through alanine scanning. In this assay, for peptide 5A, the residues that were critical for antibody recognition were proline, alanine, and aspartate. Peptide 6A did not indicate reactivity. One potential explanation for these findings is that the conformational flexibility of the peptides on the membranes may be restricted, thus reducing the affinities of the peptides for the antibody [25]. Peptide 1B showed low levels of reactivity. However, sufficient levels were present for the identification of important residues that interacted with the antibody. These results indicate that the residues of the C-terminal region were responsible for binding to the antibody. Because this portion of the sequence is immobilized in the membrane, the accessibility of the antibody to the residues is more difficult and most likely explains the low reactivities of the peptides that were derived from 1B in the membrane assay.

The peptides that were evaluated in this study were shown to be antigenic and immunogenic. This latter property allowed for the characterization of these peptides as mimotopes of *M. leprae*.

The *M. leprae* database analysis suggests that the epitope protein that is mimicked by the peptides is antigen 85B, which is a 31 kDa protein with a pI of 4.9 that possesses properties that are consistent with the information that was experimentally obtained (Fig. 4).

Antigen 85B is a component of the fibronectin-binding protein complex that presents with very interesting antigenic and immunogenic properties because of its capacity to induce strong humoral and cellular responses, including the *in vitro* proliferation of T cells [26]. Its immunological features and predominant role as a *M. leprae* antigen explain why only mimotopes of this antigen have been bioselected. These epitope-mimicking peptides contain amino acid sequences that are part of two sequences (AA 206–230 and 291–325) from three regions of antigen 85B, which have been recognized as important antigenic domains for the diagnosis of patients with lepromatous leprosy [27].

The search for antigens may not only improve the sensitivities and specificities of the diagnostic tests but also contribute to a better understanding of the immune response that is developed against the antigens in affected individuals. The results of these observations may be used to improve antigenic recognition and identify novel peptides. In addition, the peptides are very feasible for widespread use in serodiagnostic tests because they can be easily prepared in sufficient purities and large quantities [27].

This work demonstrates the usefulness of this biotechnological tool in overcoming diagnostic constraints by selecting, synthesizing, and confirming certain peptide sequences *in vitro* for their uses as diagnostic laboratory tests. The peptides that were identified were able to recognize antibodies that are primarily found in MB leprosy patients. MB cases are the principal sources of leprosy infection in the population, and affected individuals are at greater risks of developing disabilities compared with those with PB leprosy [23]. Therefore, the early detection and treatment of MB cases will contribute to a reduction in the transmission of the bacterium. Ideally, a serological test should identify all leprosy patients (MB and PB). In fact, it may be possible to diagnose all of the patients by combining peptides and assays that are based on humoral and cellular immunity. Hence, it is important to identify new peptides using the approach that is described in this work while also evaluating different diagnostic approaches using these peptides to increase the specificity and sensitivity of the detection of MB and particularly PB leprosy patients.
